# Helicobacter pylori was not detected in oral squamous cell carcinomas from cohorts of Norwegian and Nepalese patients

**DOI:** 10.1038/s41598-020-65694-7

**Published:** 2020-05-26

**Authors:** Sushma Pandey, Benoit Follin-Arbelet, Chin Bahadur Pun, Dej K. Gautam, Anne Christine Johannessen, Fernanda Cristina Petersen, Daniela Elena Costea, Dipak Sapkota

**Affiliations:** 1 Department of Oral Biology, Faculty of Dentistry, University of Oslo, Oslo, Norway; 2 Department of Pathology, B.P. Koirala Memorial Cancer Hospital, Bharatpur, Nepal; 3 Department of Surgical Oncology, B.P. Koirala Memorial Cancer Hospital, Bharatpur, Nepal; 4 Department of Clinical Medicine, The Gade Laboratory for Pathology, University of Bergen, Bergen, Norway; 5 Department of Pathology, Haukeland University Hospital, Bergen, Norway; 6 Centre for Cancer Biomarkers (CCBIO), Faculty of Medicine and Dentistry, University of Bergen, Bergen, Norway

**Keywords:** Cancer, Microbiology, Oncology

## Abstract

*Helicobacter pylori* (HP) infection is an established causative agent for gastric cancer. Although the oral cavity is a part of the gastrointestinal system, the presence and possible causative role of HP in oral squamous cell carcinoma (OSCC) is a subject of controversy. Therefore, the current study aimed to investigate HP infection in two cohorts of OSCC patients with different demographic characteristics, lifestyles and habitual risk factors. A total of 242 formalin-fixed paraffin-embedded OSCC specimens from two different patient cohorts (Norway, n = 171 and Nepal, n = 71) were used to examine HP using immunohistochemistry (IHC) and quantitative polymerase chain reaction (qPCR). Two different HP specific genes (*23S rRNA* and *ureA*) were used for TaqMan-based qPCR, and for subsequent verification using HP specific RIDAGENE HP kit and SYBR Green based qPCR. All of the OSCC specimens from both cohorts were found to be negative for HP infection with IHC and qPCR, although the positive control specimens tested positive. Our findings suggest that HP is absent in the examined OSCC cohorts, irrespective of race, lifestyle and habitual risk factors. This indicates that, in contrast to gastric cancer, HP is an unlikely contributing factor for OSCC pathogenesis.

## Introduction

Oral cancer is a highly aggressive malignant tumor arising from the anterior 2/3 of tongue, buccal mucosa, gingiva, floor of the mouth and hard palate. Oral squamous cell carcinoma (OSCC), the most common histologic variant, represents more than 90% of oral cancer cases^1^. Combined with malignancies in the lip and pharynx, oral cancer ranks as the 7^th^ most common cancer type worldwide with approximately 300 000 deaths every year^2^. Despite improvements in the diagnostic and treatment modalities, 5-year survival of OSCC patients remains around 60%^3^. Smokeless tobacco (such as betel quid, ghutka), alcohol and smoking are the most important risk factors for OSCC in Southeast Asia; whereas smoking and alcohol are important in the western countries^4,5^. Nevertheless, a fraction of OC cases cannot be linked to the aforementioned risk factors. This indicates the possibility of other factors including microorganisms in the pathogenesis of OSCC. Indeed, approximately 20% of human malignancies have been attributed to microorganisms^6^. In this context, it is worth mentioning that the oral cavity harbors approximately 700 different bacterial species^7^, and dysbiosis of several of the oral bacteria has been reported in OSCC specimens and/or brush biopsies and saliva from OSCC patients as compared to the normal controls^8–11^.

*Helicobacter pylori* (HP) is a gram negative, spiral-shaped bacterium, which is estimated to infect the stomach of almost half of the world’s population. HP was considered to be a class I carcinogen by the International Agency for Research on Cancer Working Group in 1994^12^. HP infection is considered to be the most common cause of infection-related cancers, particularly gastric cancer along with MALT lymphoma^13,14^. Elimination of HP infection is associated with the reduction in the risk of gastric cancer development from the HP infected gastric precancerous lesions, further supporting the link with gastric carcinogenesis^15^. HP can be divided into *CagA*-positive and *CagA*-negative types based on the presence or absence of *cytotoxin-associated gene A* (CagA)*. CagA*-positive HP are considered more carcinogenic than the *CagA*-negative type. At the molecular level, CagA protein (encoded by *CagA* gene) is delivered into the gastric epithelial cells by a bacterial type IV secretion system, which in turn deregulates SHP2 oncoprotein and its downstream signaling pathways, thereby contributing to the transformation of gastric epithelial cells^16^.

HP has been shown to be present in dental plaque and saliva^17^ and to colonize different parts of the oral cavity (reviewed in^18^). HP infection has been linked to several disease conditions in the oral cavity, such as stomatitis, glossitis and periodontitis^17,19^. Given the role of HP in gastric carcinogenesis and observations that HP could colonize and lead to inflammatory conditions in the oral cavity, one could hypothesize that HP infection might be related to the development of oral precancer and cancerous lesions, at least in a subset of HP positive lesions. Only a handful of studies have attempted to look at the presence of HP in OSCC, with conflicting results^20–23^. Some studies have linked the presence of HP with oral cancer progression; others have shown no significant association or even protective effect^20,22,24^. Interestingly, betel chewing has been reported to be associated with higher risk for HP infection in OSCC^21^, indicating that OSCC-related habitual risk factors might influence the susceptibly for HP infection. This study aimed to investigate HP infection status in two cohorts of OSCC specimens, one from Nepal and one from Norway, with differing race, lifestyle and habitual risk factors for OSCC, by using immunohistochemistry (IHC) and quantitative polymerase chain reaction (qPCR).

## Results

### Clinicopathological characteristics of OSCC

The clinicopathological characteristics of the specimens used are summarized in Supplementary Table S1,S2. Briefly, the median age of Nepalese OSCC cases was 56 years, and that of Norwegian cases was 66. The male to female ratio for Nepalese cases was 3:1, whereas for Norwegian cases it was 1.5:1.

### IHC

**Figure 1:**
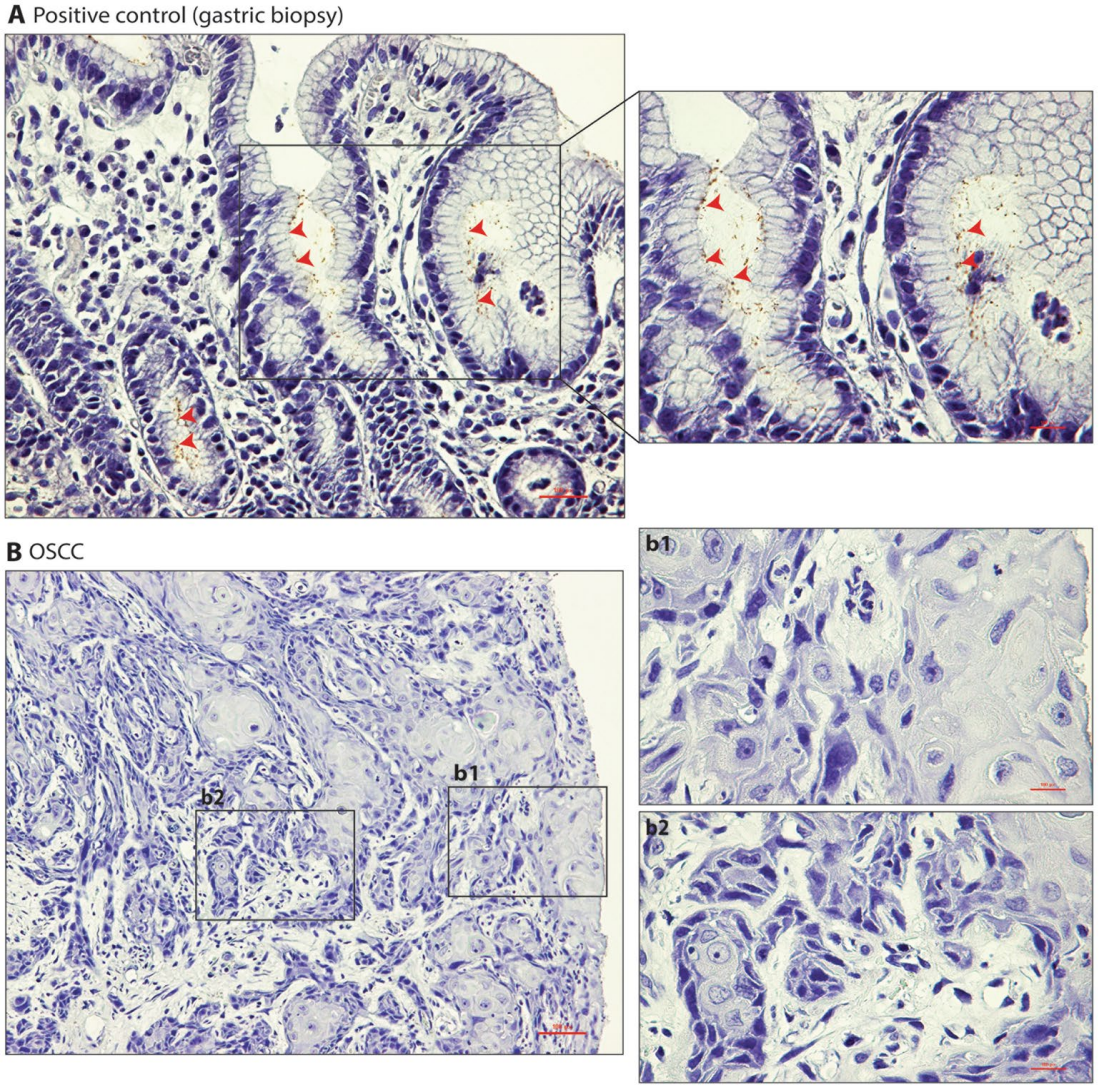
HP could not be detected in OSCC using IHC. (**A**) Representative images of gastric mucosal biopsy demonstrating presence of HP in gastric pits (red arrows). (**B**) Representative images of OSCC showing absence of HP both in the superficial (b1) and deeper parts (b2) of the lesion.

The positive control (gastric biopsies) showed brown colored rod/filament shaped HP mostly located in the gastric pits (Fig. 1A). However, all of the formalin-fixed paraffin-embedded (FFPE) OSCC specimens (n = 71) from Nepal tested negative for HP with IHC (Fig. 1B).

### DNA yield, sensitivity and amplification of TaqMan based qPCR assay

The range of DNA yield from OSCC FFPE sections was between 1.4 to 575 ng/ µl. Only 5.7% (*n* = 14) of samples yielded DNA concentration of less than 10 ng/µl. The purity (260/280 ratio) of 95% of samples (*n* = 230) was higher than 1.8.

**Figure 2:**
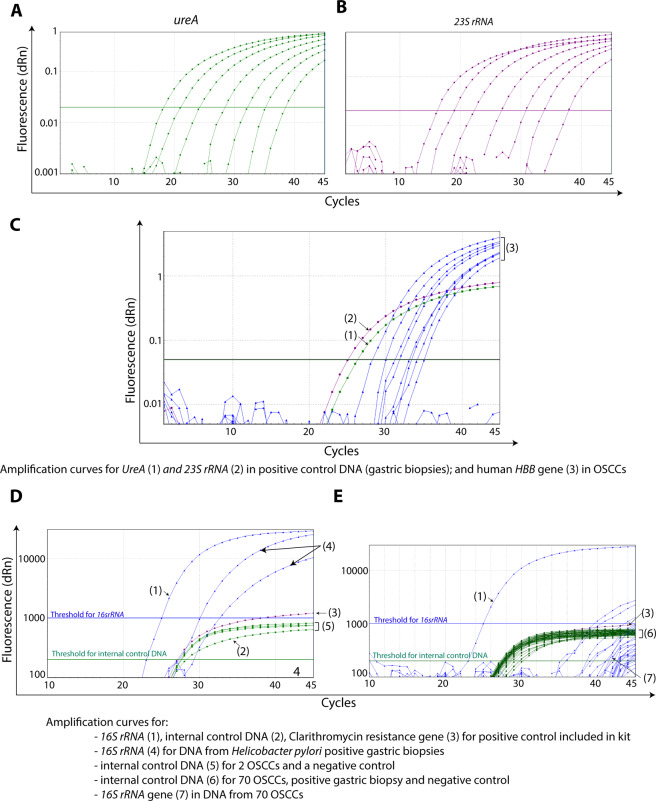
HP could not be detected in OSCC using qPCR. Representative amplification curves for HP specific *ureA* (**A**) and *23S rRNA* (**B**) genes using serial dilutions of DNA (starting concentration was 12 ng/µL) from HP culture. (**C**) Image illustrating amplification curves for HP specific *ureA* (1) and *23S rRNA* (2) genes in DNA from positive control gastric biopsy and human *HBB* (3) gene in OSCC cases. (**D** and **E**) Image illustrating amplification curves for internal control DNA gene and HP specific genes using RIDAGENE HP kit. Curves in blue color in represent amplification curves for *16S rRNA* gene for positive control (1) included in the kit and positive control gastric biopsies (4). Majority of curves in green color (labelled as 5 and 6 in figure) represent amplification curves for internal control gene in OSCC samples, positive control gastric biopsies and negative control (for details, see labels in figure).

Amplification of serially diluted DNA from positive control DNA showed that as little as 6 femtograms could be amplified by the TaqMan based assay (Fig. 2A,B). The amplification of *ureA* and *23S rRNA* was found to be 95% and 87% efficient, respectively (data not shown).

### Amplification of HP specific genes using in-house TaqMan based qPCR assay

Among the OSCC specimens (*n* = 242) from Norway and Nepal, the human *HBB* gene (coding for human beta globulin) could not be amplified in 7.02% of samples (*n* = 17), indicating poor quality of DNA and were therefore excluded from the study. All of the remaining OSCC specimens (*n* = 225) tested negative for HP specific genes with TaqMan based assay. Nevertheless, DNA from positive controls tested positive for both *23S rRNA* and *ureA* genes (Fig. 2A–C).

### Amplification of HP specific genes using *RIDAGENE HP kit*

DNA from the OSCC specimens were examined by using an independent RIDAGENE HP kit PCR. The DNA from positive controls resulted in good amplification of internal control DNA and HP specific genes (Fig. 2D,E). Despite the good amplification of internal control DNA, HP specific genes could not be amplified from DNA from OSCC samples (Fig. 2D,E).

### Amplification of HP specific genes using SYBR green-based qPCR assay

Although HP specific genes were amplified for the DNA from positive controls, none of the tested OSCC samples showed amplification for these genes (data not shown).

## Discussion

Using two different detection techniques (IHC and qPCR), the current work aimed to investigate HP infection in two different OSCCs cohorts (Norway and Nepal) differing with respect to race, lifestyle and habitual risk factors for OSCC.

Several methods (culture, serology, rapid urease test, histology/IHC, PCR) are available for the detection of HP in different types of specimens, each with some advantages and limitations^25^. The combination of qPCR and IHC was chosen in the current study as the use of these methods in FFPE specimens has been well assessed for HP detection with reasonably high sensitivity and specificity^26^. qPCR, being a highly sensitive method, is very sensitive to the quality of DNA input, well designed primer pairs and selection of proper detection chemistry^27^. To address these issues, several quality control measures were used in the current study. Firstly, all OSCC specimens with *Ct* values of >37 or not amplified for human *HBB* gene, indicating a poor quality of isolated DNA, were excluded from the study (Fig. 2C). Secondly, serial dilution series of DNA from the positive controls were used to examine the amplification efficiency (95% for *ureA* and 87% for *23S rRNA*) of the in-house TaqMan PCR system (Fig. 2A,B). Indeed, our in-house TaqMan PCR protocol was able to amplify bacterial DNA template from as little as 6 femtograms. Thirdly, commercially available RIDAGENE HP kit was used to confirm the results of in-house TaqMan qPCR. Moreover, internal control DNA included in the RIDAGENE HP kit suggested that there was no obvious inhibition to PCR amplification due to possible carry-over components in the DNA template (Fig. 2D,E). Finally, independent validation using a SYBR Green-based qPCR corroborated the results from in-house TaqMan qPCR and RIDAGENE HP kit. Similarly, proper positive and negative controls were used for IHC to ensure the validity of the method. These measures ensure that the HP test protocols in the current study were accurate and reproducible.

In the current study, none of the OSCC samples was found to be HP positive with IHC, although the positive control gastric biopsy demonstrated HP infection, primarily in the gastric pit (Fig. 1A). Supporting these results, HP specific genes (*ureA* and *23SrRNA*) were not amplified by qPCR in any of the OSCC specimens, although they were readily amplified in the positive controls (Fig. 2A–D). The current findings are in line with some previous studies reporting either an absence or lower rate of HP positivity in dental plaque, saliva or OSCC specimens, thereby questioning a causative role for HP in OSCC development^28–31^. However, another study has reported a higher rate of HP positivity (21.5%) in OSCC specimens, suggesting a link between HP and OSCC^22^. Interestingly, several other studies have found a relatively high HP seropositivity in OSCC patients as well as in healthy controls and suggested that either HP is not linked to OSCC or it can even have a protective effect on OSCC pathogenesis^20,21,24^. Overall, these observations cannot establish a role for HP in OSCC, either as carcinogenic or as a protective factor.

Several factors such as type and size of sample used, sample collection procedure, methodology used, and type of population studied might be responsible for these variable observations. Indeed, with the exception of the study by Grimm *et al*.^22^, a majority of the above-mentioned studies have used relatively smaller sample sizes. Moreover, there exists a wide variation in the sensitivities of different test methods (Supplementary Table S3). Positive serology and rapid urease test do not necessarily reflect the true HP status in biopsy specimens examined^32,33^. The current study benefited from the use of a large cohort of OSCCs and two different detection methods, with subsequent independent validation protocols. There is a wide variation in HP prevalence with respect to geography, race and lifestyle^34,35^. Betel chewing, a common habit in Southeast Asia, has been suggested to predispose for HP infection in oral cavity^21^. Absence of HP in both Nepalese and Norwegian cases with 36.6% and 0% cases with history of betel chewing respectively, suggest that betel quid might not predispose for HP infection in OSCC.

The current study has limitations. Although excluded from the analysis, about 7% OSCC cases were found to have a lower DNA quality, and availability of corresponding fresh tissues for at least for some of FFPE tissues would have been useful to examine the effect of DNA quality. The current study lacks information on the prior use of antibiotics (triple therapy), as it has been shown to eliminate HP from oral cavity in 40% of individuals^36^. In addition, oral health status^17^ as well as information on concurrent HP status in stomach^37^, considered to be important determinants for oral HP, are also unavailable. In conclusion, although oral cavity (dental plaque and saliva) has been considered as a primary extragastric reservoir for HP, the results of the current study suggest that HP is uncommon in OSCCs irrespective of race, lifestyle and habitual risk factors; and is an unlikely contributing factor for OSCC pathogenesis.

## Material and Methods

### Specimens

The tissue samples used in the current study consisted of FFPE archival OSCC specimens from Nepal (Chitwan) and Norway (Bergen). The specimens were collected respectively from B.P. Koirala Memorial Cancer Hospital (BPKMCH) (between 2011 and 2014) and Haukeland University Hospital (between 1998 and 2012). Informed consent was obtained from all participating patients. Collection of specimens from Nepal was approved by the Committee for Medical and Health Research Ethics in West Norway (2011/1244 REK vest) and Nepal Health Research Council (ref 526/2012); and specimens from Bergen by the Committee for Medical and Health Research Ethics in West Norway (2010/481 REK vest). A total number of 242 FFPE OSCCs (*n* = 71 from Nepal and *n* = 171 from Norway) were used for DNA qPCR, whereas 71 OSCCs (all from Nepal) were used for HP IHC. Protocols used for the tissue collection, processing (formalin fixation and paraffin embedding) and storage at BPKMCH were similar to that at Haukeland University Hospital. Briefly, diagnostic/surgical biopsy specimens were fixed in 10% neutral buffered formalin for up to 48 hours (depending on the size of the specimens), dehydrated using increasing ethanol concentrations and xylene, and embedded in paraffin. All methods were performed in accordance with the relevant ethical guidelines and regulations of the University of Oslo, where the experiments were performed.

### IHC

A total of 71 (*n* = 71) FFPE specimens of OSCC from Nepal (specimens from Norway were not used for HP IHC), were used for the immunohistochemical detection of HP. The details of the clinicopathological parameters of the OSCC patients are summarized in Supplementary Tables S1,S2. Four-micron thick FFPE sections were deparaffinized in xylene, rehydrated in decreasing concentrations of ethanol and antigen retrieval was done by pressure cooker treatment in Tris-EDTA buffer, pH 9.0 (Cat. no: S2367 DAKO). Blocking was done with 10% goat serum in 3% bovine serum albumin for 30 min at room temperature. Polyclonal rabbit anti-HP primary antibody (Code B047, DAKO, 1:100 dilutions) was applied for 45 minutes at room temperature. After inactivation of peroxidase by incubating with DAKO REAL (code S2023, DAKO) peroxidase blocking solution, anti-rabbit secondary antibody conjugated with horseradish peroxidase labeled polymer (EnVision System, DAKO) was applied for 30 min. Presence of antigen was visualized by staining with 3, 3′-diaminobenzidine (DAB, DAKO), counterstained with hematoxylin (DAKO) and mounted with EUKITT mounting medium. Sections incubated only with antibody diluent (Cat. no: S0809, DAKO) instead of primary antibody served as negative controls. HP positive human gastric samples, regularly used as positive controls at the diagnostic lab at the Department of Pathology, Haukeland University Hospital, were used as positive controls in the current study. IHC was evaluated by SP and DS using Nikon E90i microscope equipped with DS-Ri1 camera using the NIS-elements software.

### DNA extraction

Serially cut tissue sections (7 μm) from FFPE tissues (n = 242) were used for extraction of DNA. New microtome blade was used for sectioning every new sample. Additionally, microtome was thoroughly cleaned with DNA removal solution (DNA AWAY, Cat. no: 7010, Molecular BioProducts, USA) before and after sectioning of each paraffin block. The first paraffin section was discarded to minimize the effect of oxidized DNA on the surface of FFPE block. Several incubation times and temperatures for the treatment with proteinase K were tested and overnight incubation at 56 °C was found to be most efficient for DNA extraction from OSCC samples. Treatment with MetaPolyzyme (enzyme cocktail to break down the cell wall of bacteria, Sigma-Aldrich, Cat. no: MAC4L) was found not to improve DNA yield and amplification of HP genes in the positive control samples, and therefore was not used for DNA isolation. Following the manufacturer’s instructions, QIAamp DNA FFPE Tissue Kit (QIAGEN, cat. no: 56404) was used for DNA extraction from FFPE sections. Briefly, eight to ten consecutive sections, depending upon the size of available tissue in FFPE blocks, were placed in a 1.5 ml eppendorf tube with the help of disposable long neck pipette tips. The tissue was deparaffinized in xylene and ethanol, and digested with proteinase K overnight at 56 °C. DNA was eluted in 75 µl volume of ATE buffer. DNA concentration was measured with a NanoDrop 2000cSpectrophotometer (ND-1000, Thermo Fisher Scientific, Wilmington, DE, USA).

### Primer design and qPCR

Primer pairs and corresponding TaqMan probe sequences for two different HP specific genes (*23S rRNA*^38^, *ureA*^39^) (Table 1) were modified, and used for TaqMan- and SYBR- based qPCR systems. Primer pairs specific to human *HBB* gene (coding for Beta globulin)^40^ was used to assess the quality of isolated DNA. Serially diluted DNA isolated from HP positive gastric biopsy and HP culture (CCUG 39500 T) was used to examine the amplification efficiency, optimal reaction conditions (DNA input, primer and probe concentrations and cycling conditions) as well as used as positive controls. Samples where DNA template was replaced with an equal volume of sterile water served as negative controls.

**Table 1:** Table showing the details of primers and probes used in the study.

Target gene	primer sequence (5′ to 3′)	probe sequence (5′ to 3′)	size (bp)	reference
*ureA*	F: GCGTGGCAAGCATGATCCAT R: GGGTATGCACGGTTACGAGTTT	CAGGAAACATCGCTTCAATACCCACT	77	^39^
*23S rRNA*	F: AGGTGAAAATTCCTCCTACC R: TCTCAAGGATGGCTCCATAAG	CAAAGCCTCCCACCTATCCTGC	146	^38^
*HBB*	F: TGCCTATCAGAAAGTGGTGGCT R: GCTCAAGGCCCTTCATAATATCC	TGGCTAATGCCCTGGCCCACAA	149	^40^

bp: base pairs, F: forward; R: reverse.

### TaqMan PCR assays

Following manufacturers’ instructions, up to 200 nanograms of DNA per sample was used in a 20 μl reaction volume in 96-well PCR plate (ABgene, cat.no AB-0600) for PCR amplification of respective genes using Mx3005P qPCR system in a single replicate. Briefly, the 20 μl reaction mixture contained 10 μl of 2x Brilliant III Ultra-Fast qPCR master mix (Agilent Technologies, cat. No: 600880-51), 2 μl probe (5 µM) and primer (2,5 µM) mixture, 0,3 μl of Rox 1/500, 2,7 μl of nuclease free water and 5 µl of DNA. Master mix contained a mixture of mutant Taq DNA polymerase, dNTPs, MgCl2 (5.5 Mm) and a buffer specially formulated for fast cycling. PCR amplification was performed in Mx3005P qPCR system using the following cycling conditions: initial denaturation at 95 °C for 5 min followed by 45 amplification cycles (95 °C for 10 sec, and 60 °C for 30 sec). The data were analyzed with MxPro qPCR Software version 4.10. Samples with more than 37 Ct values were considered negative^41^.

### SYBR green PCR assays

Amplification efficiency of the primers (Table 1) were examined by using an independent master mix chemistry, SYBR green (SsoAdvanced Universal SYBR Green Supermix, BIO-RAD, cat. no: 1725271). Additionally, 9 OSCC where human *HBB* gene could not be amplified with TaqMan system, were examined using SYBR green system. The concentrations of the primers and template DNA, and the cycling conditions were same as that for TaqMan system.

### Verification with RIDAGENE HP kit

Presence of HP DNA in the OSCC samples and positive control was also examined by using RIDAGENE HP kit (cat. no: PG2305, r-biopharm). Previous studies have shown excellent sensitivity and specificity of this kit for the detection of HP in gastric biopsy^42^. This kit is based on Taq-polymerase enzyme chemistry and consists of primer pairs/probes for the HP specific genes and internal control DNA. Randomly selected OSCC samples (*n* = 60) and positive control DNA from gastric biopsy and HP culture were used for this kit. Following the manufacturer’s instructions, up to 100 nanograms of DNA template was used for PCR amplification.

## Supplementary information

Supplementary Information.

## Data Availability

The datasets generated during the current study are available from the corresponding author on reasonable request.
