# Supplementary material for: Helicobacter pylori was not detected in oral squamous cell carcinomas from cohorts of Norwegian and Nepalese patients
Source: Sci Rep. 2020 May 26;10:8737. doi: 10.1038/s41598-020-65694-7 (PMC7250879; doi:10.1038/s41598-020-65694-7)
Supplement: Supplementary file 1 — Supplementary Information. [file 41598_2020_65694_MOESM1_ESM.pdf]

Supplementary Information

Helicobacter pylori are not present in oral squamous cell carcinomas from cohorts of Norwegian and Nepalese patients

Authors:

Sushma Pandey [a*], Benoit Follin-Arbelet[a], Chin Bahadur Pun[b], Dej K. Gautam[c], Anne C. Johannessen[d,e], Fernanda Cristina Petersen[a], Daniela Elena Costea[d,e,f], Dipak Sapkota[a*]

Affiliations:

[a]*Department of Oral Biology, Faculty of Dentistry, University of Oslo, Norway;*

[b]*Department of Pathology, B.P. Koirala Memorial Cancer Hospital, Bharatpur, Nepal;*

[c]*Department of Surgical Oncology, B.P. Koirala Memorial Cancer Hospital, Bharatpur, Nepal;*

[d]*Department of Clinical Medicine, The Gade Laboratory for Pathology, University of Bergen, Norway;*

[e]*Department of Pathology, Haukeland University Hospital, Bergen, Norway;*

[f]*Centre for Cancer Biomarkers (CCBIO), Faculty of Medicine and Dentistry, University of Bergen, Norway;*

Supplementary Table S1

Clinicopathological parameters of the OSCC cases from Nepal.

Variables	Values n (%)
Age in years ¶	
≤56	36 (50.7)
>56	35 (49.3)
Gender	
Male	53 (74.5)
Female	18 (25.5)
Location	
Tongue	21 (29.5)
Gingiva /gingivolingual-/gingivobuccal-sulcus	28 (39.4)
Buccal mucosa	13 (18.3)
Floor of mouth	7 (9.8)
Alveolus	2 (2.8)
Risk habit history	
No	6 (8.4)
Yes§	65 (91.5)
Tobacco	
Smoking	44 (61.9)
Smokeless†	43 (60.5)
Betel quid‡	26 (36.6)
Alcohol	26 (36.6)
Degree of differentiation	
Well	41 (57.7)
Moderate	28 (39.4)

Poor	2 (2.8)
Clinical Staging	
Early stage (I &II)	12 (17.0)
Late stage (III & IV)	59 (83.0)

¶ Patients were categorized into low- and high-age groups based on the median age
§ Some of the patients had more than one risk habits, therefore the sum of the individual habits is more than 100%
†Most common types of smokeless tobacco products available in Nepal: raw tabacco with lime, gutkha, kaini, betel quid with tobacco
‡ Betel quid (paan) with or without tobacco

Supplementary Table S2

Clinicopathological parameters of the OSCC cases from Norway.

Variables	Values n (%)
Age¶	
≤66	86 (50.2)
>66	83 (48.5)
Missing	1(0.5)
Gender	
Male	102 (59.6)
Female	69 (40.3)
Location	
Tongue	68 (39.7)
Gingiva /gingivolingual-/ gingivobuccal-sulcus	43 (25.1)
Buccal mucosa	24 (14.0)
Floor of mouth	26 (15.2)
Others (tonsil, alveolus)	9 (5.2)
Risk habit history	
No	24(14.0)
Yes	88(51.4)
Unknown	46(26.9)
Tobacco	
Smoking	80 (46.7)
Smokeless	Not recorded
Betel quid	0 (0)
Alcohol	58 (33.9)

Degree of differentiation	
Well	80 (46.7)
Moderate	63 (36.8)
Poor	28 (16.3)
Clinical Staging	
Early stage (I & II)	82 (47.9)
Late stage (III & IV)	89 (52.0)

¶ Patients were categorized into low- and high-age groups based on the median age

Supplementary Table S3

Summary of literature on HP and OSCC/HNSCC reflecting a wide variation on the type and size of the samples used, detection methods and results.

**Author/ year**	**Sample used**	**Study design**	**Detection method(s)**	**Specimens**	**HP detection rate**	**Conclusion**
Grandis *et al* [1] (1997)	BL	CCS	Ser,<br>IgG Ab (ELISA)	21(HNSCC)<br>21 (Ctr)	Ser:12/21(HNSCC)<br>13/21(Ctr)	Lack of serologic evidence for HP infection in HNSCC.
Singh *et al*[2] (1998)	Bsy	CCS	Giemsa<br>MCT, UT	26 (OC & OU)<br>26 (Ctr)	Giemsa 4/26 (OC & OU)<br>0/26 (Ctr)<br>MCT<br>0/26 (OC & OU)<br>0/26 (Ctr)<br>UT:<br>3/26 (OC & OU)<br>0/26 (Ctr)	Absence of HP in oral mucosal lesions.
Okuda *et al*[3] (2000)	Bsy<br>OSw	PrS	MCT, PCR	116 (GD)<br>58(OC)	OSw: 14/116<br>Bsy: 14/116<br>OC: 11/58	HP may have only a transient presence in the oral cavity and on the surface of OC

Kanda *et al*[4] (2005)	Bsy Urine	PrS	MTC, PCR IHC, Ser (ELISA)	31(HNSCC)	MTC, IHC, PCR: 0/31<br>Ser: 21/31	No relationship exists between HNSCC and HP infection
Fernando *et al*[5]2009)	BL	CCS	Ser (ELISA)	53 (OC)<br>60 (Ctr)	Ser<br>14/53 (OC)<br>10/60 (Ctr)	No significant difference in HP infection was found between patients with and without oral cancer.
Dayama *et al*[6] (2011)	Bsy	CCS	MCT, PCR	20 (OC)<br>20 (Ctr)	MCT<br>3/20 (OC)<br>1/20 (Ctr)<br>PCR<br>3/20 (OC)<br>2/20 (Ctr)	No statistically significant difference in HP detection between OC and controls.
Irani *et al*[7] (2013)	Bsy	CCS	IHC	83 (OSCC)<br>32 (Ctr)	IHC:<br>69/83 (OSCC)<br>12/32 (Ctr)	HP might be a risk factor for developing OSCC.
Grimm et al[8] (2014)	Bsy CCL	CCS	Giemsa IHC	191 (OSCC)<br>10 (Ctr)	Giemsa:<br>38/191(OSCC)<br>0/10 (Ctr)<br>IHC<br>41/191(OSCC)	HP was detected in 21% of OSCCs samples HP positivity was associated with reduced disease free survival.

					0/10 (Ctr)	
Meng *et al*[9] (2016)	BL<br>Bsy	CCS	Giemsa, Ser (ELISA), PCR	68 (OC)<br>104 (Ctr)	Giemsa<br>24/68 (OC)<br>57/104 (Ctr)	HP infection may be negatively related to OSCC.

***BL**: Blood**, Bsy**: Biopsy, **OSw**: Oral swab, **CCL**: cancer cell lines, **Ctr**: Control, **CCS**: case-control study, **PrS**: Prospective Study, **Ser:** Serum, **MCT**: Microbiological cultural technique, **UT**: Urease test, **IHC**: immunohistochemistry, **HNSCC**: head and neck squamous cell carcinoma **OSCC**: oral squamous cell carcinoma, **OC**: oral cancer, **OU**: oral ulcer **HP**: helicobacter pylori*

References

1 Grandis, J. R., Perez-Perez, G. I., Yu, V. L., Johnson, J. T. & Blaser, M. J. Lack of serologic evidence for Helicobacter pylori infection in head and neck cancer. *Head Neck* **19**, 216-218 (1997).

2 Singh, K., Kumar, S., Jaiswal, M. S., Chandra, M. & Singh, M. Absence of Helicobacter pylori in oral mucosal lesions. *J Indian Med Assoc* **96**, 177-178 (1998).

3 Okuda, K. *et al.* Helicobacter pylori May Have Only a Transient Presence in the Oral Cavity and on the Surface of Oral Cancer. *Microbiol Immunol* **44**, 385-388, doi:10.1111/j.1348-0421.2000.tb02510.x (2000).

4 Kanda, T. *et al.* Investigation of helicobacter pylori in tumor tissue specimens from patients of head and neck tumor. *Practica Oto-Rhino-Laryngologica* **98**, 571-575, doi:10.5631/jibirin.98.571 (2005).

5 Fernando, N. *et al.* Presence of Helicobacter pylori in betel chewers and non betel chewers with and without oral cancers. *BMC Oral Health* **9**, 23, doi:10.1186/1472-6831-9-23 (2009).

6 Dayama, A., Srivastava, V., Shukla, M., Singh, R. & Pandey, M. Helicobacter pylori and oral cancer: possible association in a preliminary case control study. *Asian Pac J Cancer Prev* **12**, 1333-1336 (2011).

7 Irani, S., Monsef Esfahani, A. & Bidari Zerehpoush, F. Detection of Helicobacter pylori in Oral Lesions. *J Dent Res Dent Clin Dent Prospects* **7**, 230-237, doi:10.5681/joddd.2013.037 (2013).

8 Grimm, M., Munz, A., Exarchou, A., Polligkeit, J. & Reinert, S. Immunohistochemical detection of Helicobacter pylori without association of TLR5 expression in oral squamous cell carcinoma. *J Oral Pathol Med.* **43**, 35-44, doi:10.1111/jop.12082 (2014).

9 Meng, X. *et al.* An inverse association of Helicobacter pylori infection with oral squamous cell carcinoma. *J Oral Pathol Med* **45**, 17-22, doi:10.1111/jop.12324 (2016).
